# Observations on the Use of the Globe Pessary

**Published:** 1786

**Authors:** Thomas Denman

**Affiliations:** Licentiate in Midwifery of the Royal College of Physicians, Physician-man-midwife to the Middlesex Hospital, and Teacher of Midwifery in London


XII. *Obſervations on the Uſe of the Globe Peſſary. Communicated in a Letter to Dr.* Simmons, *by* Thomas Denman, *M. D. Licentiate in Midwifery of the Royal College of Phyſicians, Phyſician-man-midwife to the Middle-*

*ſex*

*sex Hospital, and Teacher of Midwifery in London.*

THE *procidentia* or *prolapsus* of the uterus is a very frequent complaint among th lower clafs of women, many of whom it renders unable to procure a livelihood by their ufual employments; and to fome it is the caufe of great mifery. I am therefore perfuaded you will excufe my requefting you to infert, in the Medical Journal, this fhort account of a peffary, not in common ufe, by which they may often be more effectually relieved, than by any other contrivance.

Many years ago, when my very worthy friend Dr. Manningham retired from bufinefs, he gave me a number of curious articles, which could not fail to be very acceptable to a perfon engaged in teaching midwifery. With thefe was a great variety of peffaries, and among the reft were fome formed of a globular piece of wood, made exceedingly thin and light by excavation. Having never before feen one of this kind, I inquired of the Doctor its hiftory, and the mode of applying it. He informed me that it was invented by the late Dr. Sandys, who had long confidered it as preferable to to any other ſort before recommended. Without giving myſelf much time for reflexion, I put this peſſary into my drawer, as a thing curious, but not likely ever to be called into uſe.

More than a year after this time, I was deſired to ſee a poor woman with a very bad *prolapſus*, which the common peſſaries were incapable of ſupporting; and revolving in my mind which would be the beſt method to purſue for the relief of my patient, I recollected Dr. Sandys' peſſaries. One of theſe was immediately introduced; ſhe has worn it for many years, and there has been no return of the complaint.

Some time after this trial, my friend Dr. Till Adams, of Briſtol, did me the favour of calling to ſhew me a globe peſſary, which had been uſed with great ſucceſs by himſelf and ſeveral other gentlemen of that city. I then ſhewed him one of Dr. Sandys' peſſaries, and gave him the preceding hiſtory of the manner in which it had come into my hands, and a detail of ſome caſes in which I had uſed it.

In the courſe of the laſt four or five years, I have met with ſeveral inſtances of patients labouring under this diſeaſe to an extreme degree, gree, who had been under the care of very able practitioners, and tried different kinds of pessaries. For all these I have used the globe pessary, and have not met with any case in which it has failed to answer my expectations. It has not only supported the uterus better than any other, but it is introduced with more facility, and is worn with more ease and comfort, not making undue pressure upon the parts on which it rests.

Being fully convinced of the utility of this pessary in my own practice, I desired Mr. Savigny, of Pall Mall, to keep some of different sizes ready for sale; by which means they are come into more frequent use, but they are not yet generally known.

To the pessaries in common use, practitioners have with great justice made many objections. But the manner in which these are introduced, is often very negligent, as if the disease was not entitled to any particular attention because it is not attended with immediate danger. Yet skill and judgement are required in the management of these cases, both previously to the introduction of a pessary, and in choosing one of a proper size. When the prolapsus has been of long continuance, and perpends perpends very low, which it ſometimes does, (even half way to the knees), the rugæ of the vagina are obliterated, and the external coat aſſumes a cuticle like that which covers any other part expoſed to the open air; and ſometimes it is tumefied by congeſtion to an enormous ſize, and ulcerated or excoriated by the flowing of the urine. In ſuch caſes it may be impoſſible to return the prolapſed uterus, till by the uſe of evacuations, of ſoothing applications, and by confinement to an horizontal poſition, it is leſſened in its ſize. Sometimes alſo the contents of the abdomen are ſo deranged, that the very reduction of ſo large a body, into a cavity now occupied by other parts, creates a temporary uneaſineſs. It will not then be expedient to introduce a peſſary, in all caſes, immediately after the reduction of the prolapſus, but to continue the fomentations, and to waſh the vagina daily with ſome aſtringent lotion injected into the vagina, or by the application of a piece of ſponge, for the purpoſe of corrugating it and favouring the reſtoration of the parts to their natural ſtate. Then the globe peſſary being introduced, the patient, after a few days farther confinement to her her bed, may by degrees return to her former occupation.

On account of ſome contingent circumſtance, objections may be made to the uſe of the globe peſſary; but for women who are not married, or who are advanced in years, however bad a prolapſus may be, I am perſuaded this peſſary will be found to deſerve the opinion I have entertained of its ſuperiority.

*Old Burlington Street,*
January 13, 1786.

